# Supplementary material for: Assessment approaches and methods in physiotherapy education: A scoping review protocol
Source: PLoS One. 2025 Oct 24;20(10):e0335229. doi: 10.1371/journal.pone.0335229 (PMC12551843; doi:10.1371/journal.pone.0335229)
Supplement: S1 Appendix — (DOCX) [file pone.0335229.s001.docx]

**MEDLINE (Ovid) run July 23, 2025 – N=2458**

Ovid MEDLINE(R) ALL <1946 to July 22, 2025>

1 Physical Therapists/ 4072

2 Physical Therapy Specialty/ 3210

3 (physiotherap* or physical therap*).tw,kf. 75889

4 or/1-3 77138

5 Educational Measurement/ 44254

6 Academic Performance/ 2303

7 Academic Success/ 2892

8 Academic Failure/ 69

9 Professional Competence/ 25456

10 Clinical Competence/ 114049

11 Computerized Adaptive Testing/ 35

12 ((assess* or evaluat* or measure* or test* or quiz* or rating* or rate* or perform* or scor* or exam* or grade* or grading or judg*) adj5 (instrument* or program* or method* or approach* or authentic* or formative or summative or evidence-based or scaffold* or competenc* or student* or design* or tool* or comprehensive or implement* or technique*)).tw,kf. 2669530

13 or/5-12 2790369

14 education/ 21682

15 academia/ 147

16 curriculum/ 93954

17 competency-based education/ 5061

18 education, professional/ 3126

19 education, continuing/ 9443

20 education, professional, retraining/ 1250

21 education, graduate/ 6308

22 schools, health occupations/ 1508

23 universities/ 59311

24 teaching/ 53303

25 models, educational/ 10790

26 patient simulation/ 5808

27 Learning/ 89502

28 ((educat* or learn* or curricul* or teach*) adj10 (universit* or college* or undergrad* or graduate or postgrad* or post grad* or "entry to practice" or academi* or clinical or accredit* or bachelor* or master* or degree* or diploma* or program* or professional)).tw,kf. 345746

29 or/14-28 582683

30 4 and 13 and 29 2462

31 remove duplicates from 30 2458
