# Supplementary material for: Assessment approaches and methods in physiotherapy education: A scoping review protocol
Source: PLoS One. 2025 Oct 24;20(10):e0335229. doi: 10.1371/journal.pone.0335229 (PMC12551843; doi:10.1371/journal.pone.0335229)
Supplement: S2 Appendix — (PDF) [file pone.0335229.s002.pdf]

Table 10: PRESS Guideline — Search Submission and Peer Review Assessment

SEARCH SUBMISSION: THIS SECTION TO BE FILLED IN BY THE SEARCHER

Searcher: Maren Goodman Email: maren.goodman@uwo.ca

Date submitted: July 28, 2025 Date requested by:

Search Topic or Title:

Assessment approaches and methods used in physiotherapy university education (any level)

This search strategy is…

My PRIMARY (core) database strategy:

This is my first submission [x] This is submitted after feedback [ ]

This is an update (the search has been previously used in an evidence syntheses) [ ]

This search strategy is…

My SECONDARY (supplemental) database strategy:

This is my first submission [ ] This is submitted after feedback [ ]

This is an update (the search has been previously used in an evidence syntheses) [ ]

Database

(e.g., MEDLINE, CINAHL, Embase): **[mandatory]**

Medline

Database Platform(s)

(e.g., Ovid, EBSCO): **[mandatory]**

Ovid

*If your chosen database or platform provides a link to the search history, please provide it here:

https://ovidsp.ovid.com/ovidweb.cgi?
T=JS&NEWS=N&PAGE=main&SHAREDSEARCHID=72MRILxrUPIeX1QBoAXcAMUQfPm9YfAEKw4m
[illegible]

Research Question(s)

(Describe the purpose of the search.) **[mandatory]**

Review objectives:
1. To identify and characterize the assessment approaches and methods used in physiotherapy education
2. To analyze the strengths, weaknesses, opportunities and threats of assessment approaches and methods used in physiotherapy education

PICO(s) or Related Format

(Outline the PICOs, SPIDER, PEPSI, etc. for your question — i.e., **P**atient, **I**ntervention, **C**omparison, **O**utcome, and **S**tudy Design — as applicable)

P	Population - Physiotherapy education
I	Concept - Assessment approaches or methods
C	Context - Occurring within an accredited university physiotherapy education program at any level (e.g., BScPT, MPT, DPT, post-graduate)
O	
S	

Inclusion Criteria

(List criteria such as age groups, study designs, and so on to be included.) **[optional]**

-Physiotherapy education at any level
-Studies describing assessment approaches or methods in the academic curriculum
-Assessments occurring within an accredited University physiotherapy education program
-Studies of any language will be included

Exclusion Criteria

(List criteria such as study designs, date limits, and so on to be excluded.) **[optional]**

-Studies will be excluded if assessment approaches or methods are examined solely to evaluate the impact or effectiveness of an educational intervention
-Assessment approaches or methods in clinical education will be excluded
-Any study design will be included with the exception of opinion pieces, commentaries and

Was a search filter applied? [mandatory]

Yes ☐ No ☒

If YES, which were used (e.g., Cochrane RCT filter, CADTH's Guidelines filter, PubMed Clinical Queries filter)? Provide the source if this is a published filter. **[mandatory if the answer was YES]**

Other notes or comments you feel would be useful for the peer reviewer (e.g., decision on date or language limits, articles used in pulling search terms)? **[optional]**

Educational measurement and related terms around assessment fall on the Education tree in MeSH, making the "assessment" and "university education" concepts tricky to delineate. We have tested excluding the term Education/ from concept 3 and accepted that it creates overlap but

Copy and paste your search strategy here, exactly as run, including the number of hits per line. **[mandatory]**

Ovid MEDLINE(R) ALL <1946 to July 22, 2025>

1 Physical Therapists/ 4072
2 Physical Therapy Specialty/ 3210
3 (physiotherap* or physical therap*).tw,kf. 75889
4 or/1-3 77138
5 Educational Measurement/ 44254
6 Academic Performance/ 2303
7 Academic Success/ 2892
8 Academic Failure/ 69
9 Professional Competence/ [illegible]

PEER REVIEW ASSESSMENT: THIS SECTION TO BE FILLED IN BY THE REVIEWER

Reviewer: Meagan Stanley, MLIS

Email: mstanle6@uwo.ca

Date completed: August 13, 2025

1. TRANSLATION

A. No revisions ☒

B. Revision(s) suggested ☐

C. Revision(s) required ☐

If "B" or "C," please provide an explanation or example:

2. BOOLEAN AND PROXIMITY OPERATORS

A. No revisions ☒

B. Revision(s) suggested ☐

C. Revision(s) required ☐

If "B" or "C," please provide an explanation or example:

3. SUBJECT HEADINGS

A. No revisions ☒

B. Revision(s) suggested ☐

C. Revision(s) required ☐

If "B" or "C," please provide an explanation or example:

4. TEXT WORD SEARCHING

A. No revisions ☒

B. Revision(s) suggested ☐

C. Revision(s) required ☐

If "B" or "C," please provide an explanation or example:

5. SPELLING, SYNTAX, AND LINE NUMBERS

A. No revisions ☒

B. Revision(s) suggested ☐

C. Revision(s) required ☐

If "B" or "C," please provide an explanation or example:

6. LIMITS AND FILTERS

A. No revisions ☒

B. Revision(s) suggested ☐

C. Revision(s) required ☐

If "B" or "C," please provide an explanation or example:

7. OVERALL EVALUATION (Note: If 1 or more of the previous elements were "revision required," this response must be "revisions required.")

A. No revisions ☒

B. Revision(s) suggested ☐

C. Revision(s) required ☐

Additional Comments (including sources of additional search terms, such as the use of text mining software or resources such as ChemID):

I asked to see portions of the protocol so I could better understand what the research question was trying to capture with "assessment". This was helpful for me as I worked through the PRESS checklist.

Please select the most appropriate answer for each element.

Element	No revisions	Revision(s) suggested	Revision(s) required
1. Translation of research question	☒	☐	☐
2. Boolean and proximity operators	☒	☐	☐
3. Subject headings	☒	☐	☐
4. Text word searching	☒	☐	☐
5. Spelling, syntax, and line numbers	☒	☐	☐
6. Limits and filters	☒	☐	☐
**Overall Evaluation**	☒	☐	☐

If revisions are suggested or required, please provide an explanation or example:

Acknowledgement

Do you wish to be acknowledged?

(If yes, the review team will be advised to add an acknowledgement to any publications related to this work).

Yes ☒ No ☐

Please use the following acknowledgement template to provide your name, postnominals, and institutional affiliation as you would like them presented.

We thank [NAME], [POSTNOMINALS (MLS, AHIP, etc.)] (Affiliated institution) for peer review of the submitted search strategy.

Meagan Stanley, MLIS, University of Western Ontario

Please use the following citation for the PRESS checklist when citing the use of PRESS in a journal publication:

Table 10: PRESS Guideline — Search Submission and Peer Review Assessment. *PRESS – Peer Review of Electronic Search Strategies: 2015 Guideline Explanation and Elaboration (PRESS E&E)*. Ottawa: CADTH; 2016 Jan. https://www.cadth.ca/press-peer-review-electronic-search-strategies-0
