# Supplementary material for: Assessment approaches and methods in physiotherapy education: A scoping review protocol
Source: PLoS One. 2025 Oct 24;20(10):e0335229. doi: 10.1371/journal.pone.0335229 (PMC12551843; doi:10.1371/journal.pone.0335229)
Supplement: Attachment — Submitted filename: Response to Academic Editor.docx [file pone.0335229.s004.docx]

**August 21, 2025**

**Response to academic editor**

Dear Jasmine Gomes,

Thank you for your rapid initial review of manuscript PONE-D-25-44509, “Assessment approaches and methods in physiotherapy education: A scoping review protocol”.

I have re-submitted this manuscript, with a request to review the decision to reject the manuscript on the basis that studies written in a language other than English would be excluded from the scoping review.

We believe that there has been a misunderstanding. The “Additional criteria” section of eligibility described a process of translating articles for consideration of eligibility. This included using Google Translate with verification of accuracy through a person proficient in the language the study is written. The final sentence of this section indicated that we will exclude studies if we cannot verify accuracy of translation. Our research team has had very good success at finding people who are proficient in languages other than English, by leveraging the resources available through the International Student Center and our multi-national graduate students. So, studies published in a language other than English are not excluded from this scoping review.

However, the study team is happy to make a minor edit to the final sentence, to better meet PLOS ONE policy: "Method of translation for each included study written in a language other than English will be reported for transparency". As the study team has only just begun title and abstract screening (<5% screened), this minor edit is feasible without impacting implementation of the protocol to date. This minor edit has been incorporated into the re-submitted protocol for consideration of publication in PLOS ONE.

Thank you for your consideration,

Katie Kowalski PT, PhD

Assistant Professor

Western University

London Ontario Canada
